# Microbiota‐related metabolites fueling the understanding of ischemic heart disease

**DOI:** 10.1002/imt2.94

**Published:** 2023-02-26

**Authors:** Yong Fan, Jiajun Ying, Hongchuang Ma, Hanbin Cui

**Affiliations:** ^1^ Key Laboratory of Precision Medicine for Atherosclerotic Diseases of Zhejiang Province Ningbo China; ^2^ Department of Cardiology, Ningbo First Hospital Ningbo University Ningbo China; ^3^ Ningbo Clinical Research Center for Cardiovascular Disease Ningbo China

## Abstract

Up‐to‐date knowledge of gut microbial taxa associated with ischemic heart disease (IHD). Microbial metabolites for mechanistic dissection of IHD pathology. Microbiome‐based therapies in IHD prevention and treatment.

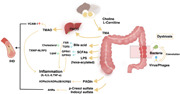

Ischemic heart disease (IHD) has caused a major burden on public health due to its high morbidity and mortality [1, 2]. IHD is commonly also referred to as coronary heart disease (CHD) or coronary artery disease, meaning a heart problem characterized by narrowed or blocked coronary arteries with reduced blood flow to the heart muscle (https://www.cdc.gov/heartdisease/coronary_ad.htm). Depending on the clinical manifestations of the disease, IHD can be classified into stable or chronic IHD and acute coronary syndrome reflecting an imbalance between myocardial oxygen demand and supply (https://www.nhs.uk/conditions/coronary-heart-disease). Patients who suffer from IHD are frequently accompanied by common risk factors for many years before overt IHD, including obesity, type 2 diabetes (T2D), and metabolic syndrome [3], calling for more specific and effective strategies for IHD prevention and intervention.

From the outcome of recent epidemiological, physiological, integrated omics‐based studies, followed by the findings from both animal and cellular investigations, it shows that a great proportion of the links between the environmental influences and human IHD may be contributed by microbial communities (termed gut microbiome) [4]. It has been revealed that the collection of all intestinal bacterial genes has more than an order of magnitude higher gene numbers than the human genome [4]. The total amount of gut bacteria exceeds 10^14^ microorganisms, whereas the gut virus has even more orders of magnitude higher quantity than that of bacteria [5]. The gut microbes, including bacteria, archea, virus, and unicellular eukaryotes, may collectively provide a repository of information characterizing the IHD development [6]. In the meanwhile, the various enzymes encoded by gut microbes may participate in pathways of producing numerous metabolites, which may via the blood circulation impact systemic and myocardial metabolism that are associated with IHD [7]. Therefore, it is reasonable to believe the strong involvement of gut microbes in the IHD development.

In the past two decades, rapid development in next‐generation sequencing technology and bioinformatic databases and tools allowed us to gain deeper knowledge of the relationships between gut microbial compositions, functional potentials, and host phenotypes, which has greatly sped up the field from cohort‐based towards personalized understanding [8]. Yet, the gap still remains between basic science and clinical translation. For instance, specific taxa is lacking for the precise diagnosis of IHD [9], the causality of microbiome on IHD is poorly understood [10], and microbiome‐based therapy in patients has not yielded satisfying efficiency [11]. Therefore, it is of great importance to explore additional mechanistic involvement of gut microbiota in the IHD development, either based on observational findings from populational studies or evidence from experimental validations. To determine the impact of gut microbiota on IHD development, the microbiota‐related metabolites may play an important and nonignorable role by mediating the alterations of microbial functionalities on host phenotypic changes.

In this review, we summarize recent advances in the IHD‐linked alterations in microbiome, not only with a focus on the taxa of bacteria (Figure 1 and Supporting Information: Table 1), but also with a particular emphasis on the microbiota‐related metabolites that regulate the initiation, escalation, and onset of IHD (Figure 2 and Table 1). We also provide insights into the updates and perspectives of microbiome‐based therapies against IHD development (Figure 3). In addition to that, we point out the gut virome/phageome as an emerging possibility in interfering the gut bacterial structure or function, thereby complementing therapeutic strategy on IHD (Figure 2).

**Figure 1:**
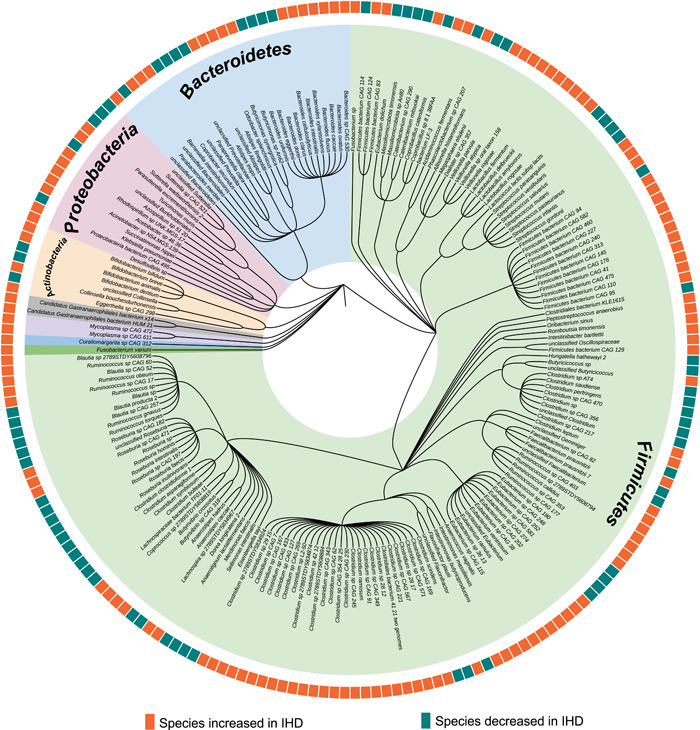
Phylogenetic tree of bacterial species associated with ischemic heart disease (IHD). This phylogenetic tree summarizes the numerous reported gut bacterial species associated with IHD. Background colors in the inner ring indicate all species that belong to the same phylum. The outer rings indicate the enrichment status of each listed bacterial species in IHD cases compared with healthy microbiota. Orange color denotes bacterial species increased in IHD cases, whereas green color denotes bacterial species decreased in IHD cases. This phylogenetic tree was created with iTOL (v6, https://itol.embl.de/).

**Figure 2:**
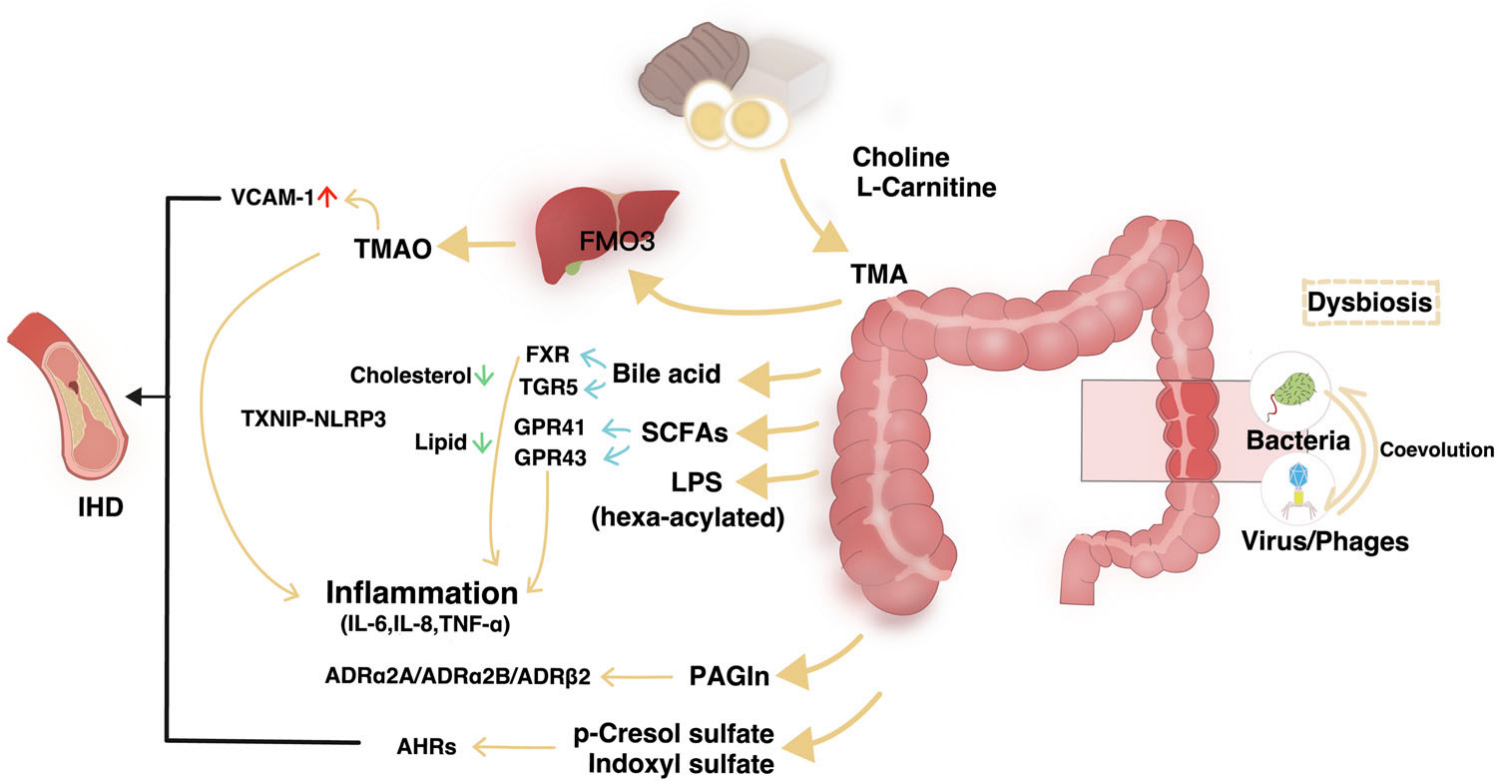
Gut‐heart axis: the potential mechanisms. An overview of some of the microbially produced compounds affecting the well‐being of cardiometabolic homeostasis during the dysbiosis associated with ischemic heart disease (IHD). Dietary l‐carnitine is metabolized by gut microbiota producing trimethylamine (TMA), which is subsequently N‐oxidized by liver flavin‐containing monooxygenases (FMOs) and producing trimethylamine N‐oxide (TMAO). TMAO has been recognized as an important contributor to atherosclerotic consequences. Secondary bile acids (BAs), synthesized from primary BAs by the intestinal microbiota, act through farnesoid X receptor (FXR) and Takeda G‐protein‐coupled receptor‐5 (TGR5) (also known as G protein‐coupled BA receptor 1 [Gpbar1]) receptors to reduce inflammation, thereby counteracting atherosclerosis. Short‐chain fatty acids (SCFAs) produced by gut microbiota through G‐protein receptor (GPR) 41/43 regulating systematic inflammation. Lipopolysaccharides (LPS), a class of pro‐inflammatory compounds, act on Toll‐like receptors, thereby activating atherosclerogenesis. Adrenoceptors have been identified as binding receptors for microbiota‐synthesized phenylacetylglutamine (PAGln). The Aryl hydrocarbon receptors (AHRs) plays crucial roles in mediating impact of two uremic toxins, p‐cresol sulfate and indoxyl sulfate, on cardiovascular system. IL, interleukin; NLRP3, NLR family pyrin domain containing 3; TNF‐α, tumor necrosis factor‐α; TXNIP, thioredoxin interacting protein; VCAM‐1, vascular cell adhesion molecule 1.

**Figure 3:**
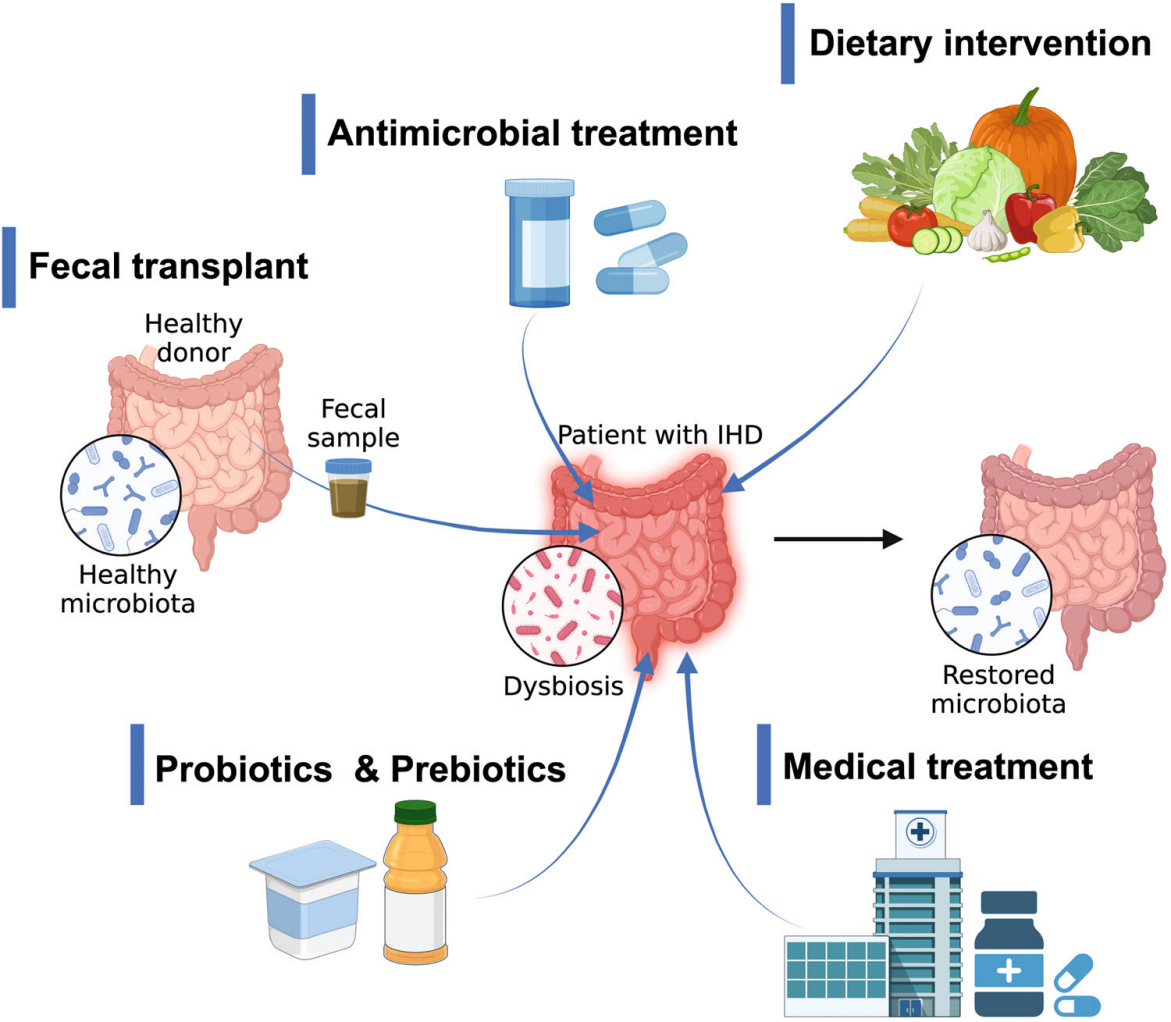
Gut microbiome‐targeted interventions in humans with ischemic heart disease (IHD). For general restoration of microbial composition and functions, there are interests in testing the microbiome‐targeted interventions on the disrupted microbiome of IHD patients. Various approaches include fecal microbiota transplantation either in heterologous or autologous manner; antibiotics treatment aiming at restructuring the gut microbiome; individualized nutrition to change the gut microbiome and metabolism; use of probiotics and prebiotics or combination of various probiotics strains and prebiotics; and finally, an emerging potential frontier of using drugs targeting specific IHD‐related microbial metabolites or pathways. Illustration was created with biorender (https://biorender.com/).

## GUT BACTERIAL CHANGES ASSOCIATED WITH IHD DEVELOPMENT

Patients with different types of IHD are often found to be associated with gut dysbiosis at multiple resolutions (Figure 1). However, due to the fact that patients with atherosclerosis are frequently found to have prior clinically silent metabolic dysregulations for many years, it is therefore a major challenge to delineate the putative impact of gut bacterial imbalance on early‐stage metabolic dysfunction from IHD onset. In addition to that, most of the IHD patients are always heavily medicated on its various comorbidities, such as obesity, T2D, and so on, making it even more challenging to decode the gut microbial alterations directly linked to IHD itself without accounting for the dysbiosis induced by its premorbidities and comorbidities. In 2017, Cui et al. [12] found that the abundance of phylum *Bacteroidetes* and *Proteobacteria* in patients with IHD were lower than that in the controls without adjusting for any medications or comorbidities. In addition, the class *Bacteroidia*, belonging to phylum *Bacteroidetes*, was significantly decreased in the IHD patient group compared with the control group. In contrast, phylum *Firmicutes* and *Fusobacteria* was higher than that in the controls [12]. At the family level, in 2019, Liu et al. [13] reported that *Lachnospiraceae* and *Ruminococcaceae* decreased significantly in IHD patients. Meanwhile, they found that Proteobacteria phylotypes such as *Streptococcus*, *Haemophilus*, and *Granulicatella* increased higher in the group with more severe heart disease by multiple comparisons among its subgroups [13]. In addition, they found that the abundance of the co‐abundance group 17, which contained several Gram‐negative bacteria, such as *Veillonella*, *Haemophilus*, and *Klebsiella*, increased with IHD severity [13]. According to the previous findings, these bacteria trigger the innate immune response via lipopolysaccharide (LPS) production and elicit a subsequent inflammatory reaction [14]. At the genus level, Jie et al. [9] found that there was a relative reduction in *Bacteroides* and *Prevotella*, and enrichment in *Streptococcus* and *Escherichia* in gut bacteriome of patients with atherosclerotic cardiovascular disease (ACVD). The abundance of *Enterobacteriaceae* and the bacteria that are often found in the oral cavity, such as *Streptococcus* spp., *Lactobacillus salivarius*, *Solobacterium moorei*, and *Atopobium parvulum*, were also higher in patients with ACVD than in healthy controls. In contrast, butyrate‐producing bacteria including *Roseburia intestinalis* and *Faecalibacterium prausnitzii* were depleted in the ACVD gut bacteriome. It is worthwhile to note that the gut microbiota also showed differences in network structure between ACVD and healthy individuals. For instance, it was found that ACVD microbiome is characterized by a negative correlation between ACVD‐depleted commensals *Bacteroides* spp. and aerobes *Streptococcus* spp., which is intriguingly absent in normal control gut bacteriome. These results demonstrated profound imbalances in the composition and inter‐species relationship in the gut microbiome of ACVD patients as compared with healthy controls [9].

Of special interest is the impact of major disease confounders on IHD microbiome analysis, including promorbidities, comorbidities, and multidrug interventions, which have gained attention. A recent work focused on characterizing the altered microbial features along the nature history of IHD, including disease initiation and escalation, while accounting for the effects of medication and lifestyle, on different IHD stages. It was reported long before the early clinical manifestation of IHD, the major microbial and metabolic alterations had already begun. The researchers additionally by using machine learning algorithms identified deconfounded IHD‐specific microbiome and metabolome features, which likely provide better capacity in IHD subgroup classification than that of the conventional IHD biomarkers. The IHD‐specific bacterial features are composed of 23 species including *Acinetobacter*, *Turcimonas*, and *Acetobacter* depleted in IHD patients, and 8 species enriched in IHD which contains 2 species in Burkholderiales order [10]. One of the two species in Burkholderiales order was reported as a possible cause of endocarditis [15]. This work highlighted the importance of accounting for interactions by confounders when analyzing microbiome data in complex noncommunicable diseases. In another Israeli cohort, Yeela et al. [16] reported that 20 bacterial genomes significantly enriched in either the patients with acute coronary diseases (ACS) or the control individuals by adjusting the confounders including clinical parameters and multidrug usage. They found that butyrate‐producing bacteria such as *Clostridium*, *Anaerostipes hadrus*, *Streptococcus thermophilus*, and *Blautia* decreased, whereas the abundance of *Odoribacter splanchnicus* and *Escherichia coli* increased in ACS patients. In addition to known bacterial features, they found a previously unknown bacterial species of the *Clostridiaceae* family that was depleted in ACS [16]. Interestingly, researchers from both groups found that butyrate producers decreased in IHD patients, which may lead to the reduced production potential of short‐chain fatty acids (SCFAs). As for acute myocardial infarction (AMI), Han et al. [17] recruited 30 in‐hospital AMI patients in China and found bacteria belonging to the phyla *Actinobacteria*, *Cyanobacteria*, *Proteobacteria*, and *Verrucomicrobia* are enriched in AMI gut microbiome, whereas the phyla Fusobacteria and Tenericutes decreased. In addition, they reported that the patients who suffered the AMI caused by left anterior descending coronary stenosis are characterized by enriched *Ruminantium* group, *Comamonadaceae*, *Comamonas*, and unknown species belonging to the MollicutesRF9 order [17]. This study, although without considering the confounding effects of polypharmacy and lifestyle in a relatively small cohort, points to the potential for gut microbial involvement in AMI caused by coronary branch vessel stenosis.

## MICROBIOTA‐RELATED METABOLITES FUEL THE MECHANISTIC UNDERSTANDING OF GUT BACTERIA IN IHD

Vast studies have revealed that the compositional and structural aberrancies in gut microbiota characterize IHD patients. However, the underlying mechanistic information of microbiota‐IHD associations remains unsystematically reviewed. In fact, the gut microbiome, as a genetic repository, is an immense factory that can release or synthesize overwhelming numbers of chemicals needed for the communication between gut commensals and host (Table 1). In the following section, we selectively summarize the IHD‐specific bacterial messengers and their impacts on IHD pathophysiology (Figure 2).

**Table 1:** Bacterial producers of microbiota‐related metabolites and their signaling pathways involved in IHD.

Human gut bacterial producers [18, 19, 20, 21, 22, 23]	Gut microbiota‐related metabolites	Potential receptors and molecular pathways linking gut microbial metabolites to IHD
p_*Actinobacteria*|c_Not assigned|o_*Coriobacteriales*|f_*Coriobacteriaceae*|g_*Collinsella*|s_*Collinsella aerofaciens*	Trimethylamine N‐oxide	Receptors: TAAR5 [24]; PERK [25], and so on
p_*Bacteroidetes*|c_*Bacteroidia*|o_*Bacteroidales*|f_ *Bacteroidaceae*|g_*Bacteroides*|s_*Bacteroides caccae*		
p_*Bacteroidetes*|c_*Bacteroidia*|o_*Bacteroidales*|f_ *Bacteroidaceae*|g_*Bacteroides*|s_*Bacteroides ovatus*		Pathways: Atherosclerosis [26]; Thrombosis [27]; Inflammatory regulation [28]; Cardiorenal fibrosis [29], and so on
p_*Bacteroidetes*|c_*Bacteroidia*|o_*Bacteroidales*|f_ *Bacteroidaceae*|g_*Bacteroides*|s_*Bacteroides thetaiotaomicron*		
p_*Firmicutes*|c_*Clostridia*|o_*Clostridiales*|f_ *Clostridiaceae*|g_*Clostridium*|s_*Clostridium asparagiforme*		
p_*Firmicutes*|c_*Clostridia*|o_*Clostridiales*|f_ *Clostridiaceae*|g_*Clostridium*|s_*Clostridium hathewayi*		
p_*Firmicutes*|c_*Clostridia*|o_*Clostridiales*|f_ *Clostridiaceae*|g_*Clostridium*|s_*Clostridium sporogenes*		
p_*Firmicutes*|c_*Clostridia*|o_*Clostridiales*|f_*Eubacteriaceae*|g_*Eubacterium*|s_*Eubacterium rectale*		
p_*Firmicutes*|c_*Clostridia*|o_*Clostridiales*|f_Not assigned|g_*Anaerococcus*|s_*Anaerococcus hydrogenalis*		
p_*Proteobacteria*|c_*Gammaproteobacteria*|o_*Enterobacteriales*|f_*Enterobacteriaceae*|g_*Edwardsiella*|s_*Edwardsiella tarda*		
p_*Proteobacteria*|c_*Gammaproteobacteria*|o_*Enterobacteriales*|f_*Enterobacteriaceae*|g_*Escherichia*|s_*Escherichia fergusonii*		
p_*Proteobacteria*|c_*Gammaproteobacteria*|o_*Enterobacteriales*|f_*Enterobacteriaceae*|g_*Proteus*|s_*Proteus penneri*		
p_*Proteobacteria*|c_*Gammaproteobacteria*|o_*Enterobacteriales*|f_*Enterobacteriaceae*|g_*Providencia*|s_*Providencia rettgeri*		
p_*Bacteroidetes*|c_*Bacteroidia*|o_*Bacteroidales*|f_ *Bacteroidaceae*|g_*Bacteroides*|s_*Bacteroides fragilis*	Short‐chain fatty acids	Receptors: GPR41/43 [30]; Olfr78 [31], and so on
p_*Bacteroidetes*|c_*Bacteroidia*|o_*Bacteroidales*|f_ *Bacteroidaceae*|g_*Bacteroides*|s_*Bacteroides thetaiotaomicron*		Pathways: Inflammatory modulation [32]; Myocardial regeneration [33]; Blood pressure homeostasis [34], and so on
p_*Firmicutes*|c_*Negativicutes*|o_*Selenomonadales*|f_*Acidaminococcaceae*|g_*Phascolarctobacterium*|s_*Phascolarctobacterium succinatutens*		
p_*Firmicutes*|c_*Negativicutes*|o_*Selenomonadales*|f_*Veillonellaceae*|g_ *Dialister*|s_*Dialister succinatiphilus*		
p_*Firmicutes*|c_*Negativicutes*|o_*Selenomonadales*|f_*Veillonellaceae*|g_ *Veillonella*|s_*Veillonella parvula*		
p_*Firmicutes*|c_*Negativicutes*|o_*Selenomonadales*|f_*Veillonellaceae*|g_*Megasphaera*|s_*Megasphaera elsdenii*		
p_*Firmicutes*|c_*Negativicutes*|o_*Selenomonadales*|f_*Veillonellaceae*|g_*Selenomonas*|s_*Selenomonas ruminantium*		
p_*Actinobacteria*|c_Not assigned|o_*Bifidobacteriales*|f_*Bifidobacteriaceae*|g_*Bifidobacterium*|s_*Bifidobacterium adolescentis*	Bile acids	Receptors: TGR5 [35]/FXR [36]/LXR [37], and so on
p_*Actinobacteria*|c_Not assigned|o_*Bifidobacteriales*|f_*Bifidobacteriaceae*|g_*Bifidobacterium*|s_*Bifidobacterium bifidum*		
p_*Actinobacteria*|c_Not assigned|o_*Bifidobacteriales*|f_*Bifidobacteriaceae*|g_*Bifidobacterium*|s_*Bifidobacterium longum*		
		Pathways: Lipid metabolism regulation [38]; Glucose/Insulin homeostasis [39]; Immunity [40], and so on
p_*Bacteroidetes*|c_*Bacteroidia*|o_*Bacteroidales*|f_ *Bacteroidaceae*|g_*Bacteroides*|s_*Bacteroides fragilis*		
p_*Bacteroidetes*|c_*Bacteroidia*|o_*Bacteroidales*|f_ *Bacteroidaceae*|g_*Bacteroides*|s_*Bacteroides vulgatus*		
p_*Firmicutes*|c_*Bacilli*|o_*Bacillales*|f_*Listeriaceae*|g_*Listeria*|s_*Listeria monocytogenes*		
p_*Firmicutes*|c_*Bacilli*|o_*Lactobacillales*|f_*Lactobacillaceae*|g_*Lactobacillus*|s_*Lactobacillus acidophilus*		
p_*Firmicutes*|c_*Bacilli*|o_*Lactobacillales*|f_*Lactobacillaceae*|g_*Lactobacillus*|s_*Lactobacillus johnsonii*		
p_*Firmicutes*|c_*Bacilli*|o_*Lactobacillales*|f_*Lactobacillaceae*|g_*Lactobacillus*|s_*Lactobacillus plantarum*		
p_*Firmicutes*|c_*Clostridia*|o_*Clostridiales*|f_ *Clostridiaceae*|g_*Clostridium*|s_*Clostridium perfringens*		
p_*Bacteroidetes*|c_*Bacteroidia*|o_*Bacteroidales*|f_ *Bacteroidaceae*|g_*Bacteroides*|s_*Bacteroides caccae*	Lipopolysaccharide	Receptors: TLR2/4 [41]/9 [42], and so on
p_*Bacteroidetes*|c_*Bacteroidia*|o_*Bacteroidales*|f_ *Bacteroidaceae*|g_*Bacteroides*|s_*Bacteroides dorei*		
p_*Bacteroidetes*|c_*Bacteroidia*|o_*Bacteroidales*|f_ *Bacteroidaceae*|g_*Bacteroides*|s_*Bacteroides fragilis*		Pathways: Proinflammation [42]; Vascularly functional homeostasis, and so on
p_*Bacteroidetes*|c_*Bacteroidia*|o_*Bacteroidales*|f_ *Bacteroidaceae*|g_*Bacteroides*|s_*Bacteroides ovatus*		
p_*Bacteroidetes*|c_*Bacteroidia*|o_*Bacteroidales*|f_ *Bacteroidaceae*|g_*Bacteroides*|s_*Bacteroides uniformis*		
p_*Bacteroidetes*|c_*Bacteroidia*|o_*Bacteroidales*|f_ *Bacteroidaceae*|g_*Bacteroides*|s_*Bacteroides vulgatus*		
p_*Bacteroidetes*|c_*Bacteroidia*|o_*Bacteroidales*|f_*Prevotellaceae*|g_*Prevotella*|s_*Prevotella copri*		
p_*Actinobacteria*|c_Not assigned|o_*Coriobacteriales*|f_ *Coriobacteriaceae*|g_*Collinsella*|s_ *Collinsella intestinalis*	Phenylacetylglutamine	Receptors: ADRA2A/ADRA2B/ADRB2 [43], and so on
p_*Actinobacteria*|c_Not assigned|o_*Coriobacteriales*|f_ *Coriobacteriaceae*|g_*Collinsella*|s_*Collinsella aerofaciens*		
p_*Bacteroidetes*|c_*Bacteroidia*|o_*Bacteroidales*|f_ *Bacteroidaceae*|g_*Bacteroides*|s_*Bacteroides caccae*		
p_*Bacteroidetes*|c_*Bacteroidia*|o_*Bacteroidales*|f_ *Bacteroidaceae*|g_*Bacteroides*|s_*Bacteroides cellulosilyticus*		Pathways: Atherosclerosis [43]; Thrombosis [43], and so on
p_*Bacteroidetes*|c_*Bacteroidia*|o_*Bacteroidales*|f_ *Bacteroidaceae*|g_*Bacteroides*|s_*Bacteroides ovatus*		
p_*Bacteroidetes*|c_*Bacteroidia*|o_*Bacteroidales*|f_ *Bacteroidaceae*|g_*Bacteroides*|s_*Bacteroides thetaiotaomicron*		
p_*Bacteroidetes*|c_*Bacteroidia*|o_*Bacteroidales*|f_ *Bacteroidaceae*|g_*Bacteroides*|s_*Bacteroides uniformis*		
p_*Bacteroidetes*|c_*Bacteroidia*|o_*Bacteroidales*|f_ *Rikenellaceae*|g_ *Alistipes*|s_*Alistipes indistinctus*		
p_*Firmicutes*|c_*Bacilli*|o_*Bacillales*|f_ *Staphylococcaceae*|g_*Staphylococcus*|s_*Staphylococcus aureus*		
p_*Firmicutes*|c_*Clostridia*|o_*Clostridiales*|f_ *Clostridiaceae*|g_*Clostridium*|s_*Clostridium asparagiforme*		
p_*Firmicutes*|c_*Clostridia*|o_*Clostridiales*|f_ *Clostridiaceae*|g_*Clostridium*|s_*Clostridium hathewayi*		
p_*Bacteroidetes*|c_*Bacteroidia*|o_*Bacteroidales*|f_*Muribaculaceae*|g_*Barnesiella*|s_*uncultured Barnesiella sp*.	p‐Cresol sulfate, Indoxyl sulfate	Receptors: AHRs [44], and so on
p_*Bacteroidetes*|c_*Bacteroidia*|o_*Bacteroidales*|f_*Muribaculaceae*|g_*Duncaniella*|s_*uncaniella dubosii*		Pathways: Kidney loss of function [45]; ROS production [46], proinflammation [47], and so on
p_*Bacteroidetes*|c_*Bacteroidia*|o_*Bacteroidales*|f_*Muribaculaceae*|g_*Muribaculum*|s_*uncultured Muribaculum sp*.		
p_*Bacteroidetes*|c_*Bacteroidia*|o_*Bacteroidales*|f_*Muribaculaceae*|g_Unknown|s_*uncultured Muribaculaceae bacterium sp*.		
p_*Bacteroidetes*|c_*Bacteroidia*|o_*Bacteroidales*|f_*Odoribacteraceae*|g_*Culturomica*|s_*Culturomica sp*.		
p_*Firmicutes*|c_*Clostridia*|o_*Clostridiales incertae sedis*|f_*Clostridiales family XIII Incertae Sedis*|g_*Aminipila*|s_*Aminipila butyrica*		
p_*Firmicutes*|c_*Clostridia*|o_*Clostridiales*|f_*Lachnospiraceae*|g_*Anaerobium*|s_*Anaerobium sp*.		
p_*Proteobacteria*|c_*Betaproteobacteria*|o_*Burkholderiales*|f_*Sutterellaceae*|g_*Turicimonas*|s_*Turicimonas muris*		

Abbreviations: ADR, adrenoceptor; AHR, acryl hydrocarbon receptor; c_, class; f_, family; FXR, farnesoid X receptor; g_, genuss; GPR41/43, G‐protein‐coupled receptor 41/43; IHD, ischemic heart disease; LXR, liver X receptor; o_, order; Olfr78, olfactory receptor 78; p_, phylum; PERK, protein kinase R (PKR)‐like endoplasmic reticulum kinase; ROS, reactive oxygen species; s_, species; TAAR5, trace amine‐associated receptor 5; TGR5, G‐protein‐coupled bile acid receptor 1; TLR2/4/9, Toll‐like receptor 2/4/8.

### Production of trimethylamine‐N‐oxide (TMAO)

Dietary factors such as choline and carnitine are closely related to TMAO, which is proved as an independent risk factor for IHD [48, 49, 50]. TMAO comes from many sources, such as egg, fish, red meat, and so on [51]. In 2013, Hazen and colleagues [48] found that TMAO was an independent risk factor for IHD and subsequent experiments demonstrated that TMAO levels were associated with death and nonfatal myocardial infarction [48, 52, 53]. The precursor of TMAO is trimethylamine (TMA), which is produced by intestinal microorganisms from nutrients containing l‐carnitine or phosphatidylcholine [50]. TMA produced by intestinal microorganisms can enter the host circulation and reach hepatocytes. In the liver, kidney, and other tissues, TMA is metabolized by flavin‐containing monooxygenase (FMO), which is encoded by FMO gene [54, 55]. Higher production of TMAO will affect lipid metabolism and reduce cholesterol clearance by inhibiting the synthesis of bile acids (BAs) [56, 57]. This may be because TMAO induces the expression of two scavenging receptors (CD36 and scavenger receptor A) on the cell surface which lead to inhibit the reverse transport of cholesterol and the accumulation of cholesterol in macrophages [58]. Moreover, TMAO can also induce calcium release and platelet hyperreactivity, thereby affecting the IHD development [59]. TMAO can upregulate inflammatory factors such as tumor necrosis factor α (TNF‐α), interleukin (IL)‐6, IL‐18 through the activation of TXNIP‐NLRP3 [60]. It can boost the expression of vascular cell adhesion molecule 1 and monocyte adhesion, which can lead to plaque development [61].

The gut microbial composition is a major factor that impacts TMAO production. Gwen's study identified a total of 102 genomes from 36 species classified as *Firmicutes*, *Proteobacteria*, and *Actinobacteria* [51] that influenced the production of TMA. Another study found that eight species representing phylum Firmicutes and Proteobacteria, and six genera consume more than 60% of choline presented in the media, which subsequently led to a remarkable production of TMA [18, 62]. Additional experiments have also expanded the TMA‐producing bacteria to *Ruminococcus* [63]. In addition, the taxonomic identification of TMA‐producing gut bacteria, biosynthetic genes, and gene clusters (BGCs) responsible for the production TMA have been reported. Two dominant TMA synthesis pathways have been extensively studied; these are as follows: (1) using choline as a substrate via the choline TMA‐lyase (CutC) and its activator CutD [64]; (2) acting on carnitine through a two‐component Rieske‐type oxygenase/reductase (CntA/B) [65]. In addition, the enzyme complex YeaW/X has also been shown to take part in the TMA synthesis [66]. The γ‐butyrobetaine (γBB)‐specific BGCs are six adjacent genes consisting of one acyl‐CoA dehydrogenase (gbuA), two acyl‐CoA transferases (gbuB, gbuC), a ubiquinone oxidoreductase (gbuD), a betaine/carnitine/choline transporter (gbuE), and one acyl‐CoA thioester hydrolase (gbuF), among which four genes were identified as necessary and sufficient for TMA production in the non‐native *E. coli* host: gbuA, gbuB, gbuC, and gbuE [67].

Although TMAO is the most widely studied independent risk factor related to IHD, more microbiota‐dependent risk factors have been found. For instance, trimethyllysine (TML), a precursor of the synthesis of carnitine, which can be metabolized to proatherogenic TMA, is a strong predictor of incident IHD, independent of TMAO [68]. Another proatherogenic agent, γBB, which is an intermediate in gut microbial transformation of carnitine to TMA, was also found to be closely related to the risk of IHD in clinical cohort (*n* = 2918). Furthermore, *N*,*N*,*N*‐trimethyl‐5‐aminovaleric acid (TMAVA), which was derived from TML through the gut microbial metabolism, was elevated with gradually increased risk of cardiac mortality and transplantation in a prospective heart failure cohort (*n* = 1647) [69]. Zhao et al. [69] found that TMAVA increased significantly, especially in patients with hypertension, which may lead to cardiac hypertrophy. In addition, they supplemented mice on a high‐fat diet for 12 weeks. They discovered that heart weight was increased in the TMAVA‐treated mice, compared with the untreated controls, which suggested that TMAVA aggravates cardiac hypertrophy and dysfunction induced by heart failure. They also found that TMAVA treatment leads to myocardial lipid accumulation and carnitine reduction in plasma and myocardium. They supposed that TMAVA functions through γBB hydroxylase (BBOX) by mice experimenting with BBOX deficiency [69].

In conclusion, TMAO and other related precursors in its synthetic pathway, play an important role in the occurrence and development of IHD, and relevant pathways remain to be further explored.

### Synthesis of SCFAs

SCFAs including acetate, propionate, and butyrate are fermented from monosaccharides and are the main bacterial products [70, 71]. Acetate and propionate are mostly produced by the phylum Bacteroidetes, whereas butyrate is mainly produced by the phylum Bacteroidetes and Firmicutes [72, 73]. Research by Jie et al. [9] showed that the gut microbiome of IHD patients is characterized by a reduction in *Roseburia* and *Eubacterium*, two known producers of butyrate. Consistently, they found the functional potential for butyrate production reduced in IHD patients [9]. SCFAs have positive effects including regulating intestinal pH, decreasing body weight, improving insulin sensitivity, and promoting intestinal motility [73, 74]. The SCFAs can also reduce blood lipid levels by transferring cholesterol to the liver and blocking cholesterol synthesis [75]. SCFAs are transported by specific monocarboxylate transporters through the intestine into the blood. SCFAs work by acting as a ligand for the G‐protein‐coupled receptors (GPR43 and GPR41) [76, 77]. These receptors play a vital role in the regulation of energy consumption and expenditure, and immune response. Furthermore, another research reported that there was a strong negative correlation between butyrate‐producing genes and C‐reactive protein (CRP) levels [78], which has been reported to be closely related to the occurrence of IHD.

The gut SCFA‐producing bacteria have been shown to be less abundant in certain IHD and hypertension patients [78, 79]. Both SCFA‐producing bacterial features and SCFAs are considered as a protective element in IHD development. Thus, targeted strategies enriching the SCFAs and its producers are potential therapeutic means for IHD preventions.

### BA modulation

Gut microbiota is one of the main contributors in regulating circulating BAs [80, 81]. Primary BAs are synthesized by the oxidation of cholesterol in the liver and secreted into the intestine as taurine‐ or glycine‐conjugated forms at C24 to dissolve lipids for absorption through the rate‐limiting enzyme cholesterol 7‐α‐hydroxylase (CYP7A1) [82]. Primary BAs (cholic acid and chenodeoxycholic acid) are converted into secondary BAs (deoxycholic acid, lithocholic acid [LCA], ursodeoxycholic acid [UDCA], and so on) through microbial dehydroxylation [83]. About 95% of BAs are reabsorbed and recycled from the intestine, except for LCA and UDCA [84]. BAs can act as ligands activating nuclear receptor farnesoid X receptor and Takeda G‐protein‐coupled receptor‐5 (TGR5) [82, 85]. Through activating the two receptors, BAs can reduce the serum cholesterol level [86]. Moreover, upon activation of TGR5, BAs can also protect LPS‐induced inflammation [87]. More specifically related to IHD development, the study led by Mayerhofer et al. [88] demonstrated that BAs reduce heart rate and regulate vascular tension via regulating channel conductance and calcium dynamics. Moreover, they found that the primary to secondary BAs ratio is positively correlated with the level of circulating cholesterol in patients with heart failure and IHD development [88]. Although most studies focus on describing the associations between gut microbes and circulating BAs, a deeper understanding towards the regulator of gut microbiota responsible for BAs metabolism is sparse. As a notable example, Wang et al. [89] demonstrated the gut bacterial structural variations (SVs) greatly determine the BAs metabolism. Systematically characterizing two types of SVs, deletion and variable SVs, in the human gut microbiome from two cohorts consisting of 1437 participants, and associating the SVs profile to circulating BAs, allowed the investigators to identify the genetic regions in specific bacterial genomes that are responsible for BAs regulation. More interestingly, such a strategy also identifies putative regions encoding BA‐metabolizing enzymes, although experimental evidence is still lacking due to the big challenge in isolating bacterial strains carrying the identified regions.

We assume that if the gut microbial features (taxas, functional potentials, and SVs), which is related to BAs metabolism, are in a state of imbalance, IHD is developed. Therefore, the BA‐relevant bacterial functions, receptors, and pathways need to be explored and may be targeted for therapeutic intervention of IHD.

### LPS and immune regulation

The depletion of butyrate‐producing bacteria may not only cause reduction of butyrate but also lead to intestinal mucosal barrier dysfunction and increase the passive leakage of microbial toxins, such as LPS and other receptors of the innate immune system, leading to inflammation [90, 91]. Recently, Awoyemi et al. [92] reported that increasing levels of LPS‐binding protein associated with high risk of IHD. Intestinal leakage may also lead to the translocation of LPS [93]. Several studies have reported that hexa‐acylated LPS but not penta‐acylated LPS can lead to systematic inflammation [94, 95]. Therefore, we highlight that hexa‐acylated LPS may be a potential target IHD treatment.

Gut microbiota can lead to IHD development via regulating our immune system. IHD is a chronic inflammatory disease, whereas AMI is suspected to be associated with acute inflammation [96, 97]. In our body, oxidized low density lipoprotein (oxLDL) can promote atherosclerosis and inflammation by activating endothelial cells, macrophages, and T cells. Macrophages can promote generation of inflammatory factors (TNF‐α, IL‐6, IL‐18, and IL‐37) by devouring oxLDL and leading to IHD development as a consequence [98, 99]. The composition of gut microbiota can strongly influence body's immune system. The study by Mikelsaar et al. [100] reported that the quantity of *Lactobacillus reuteri*, which exists in the intestine, is associated with high levels of white blood cells. Furthermore, Low *Oscillibacter*, *Faecalibacterium*, and *Ruminococcus* are correlated with high CRP level [101, 102]. Besides, germ‐free mouse models showed that the development of T cells is directly influenced by gut microbiota, particularly the differentiation of T helper 17 cells (Th17) [103, 104, 105]. The research by Gil‐Cruz et al. [60] reported that myocarditis may depend on specific Th17 cells derived from gut microbiota. They additionally found that *Bacteroides thetaiotaomicron* and *B. faecis* can promote inflammatory myocardiopathy [60]. Furthermore, butyrate produced by gut bacteria promotes the forkhead box P3 (Foxp3+) regulatory T cell induction [106], as well as acts on the GPR43 and GPR41 for affecting immune system [76, 107].

### Phenylacetylglutamine (PAGln) production

PAGln, a product from microbial fermentation of dietary phenylalanine followed by conjugation to glutamine, has been reported to be associated with IHDs and major adverse cardiovascular events independently [108]. Among patients with carotid plaque, plasma level of PAGln was significantly lower in protected phenotype rather than other more severe phenotype. Therefore, it is considered that lower PAGln may contribute to plaque stability in carotid atherosclerosis [109]. As for patients with IHD, Liu et al. [110] reported an independent association between plasma PAGln levels and the coronary atherosclerotic burden. Patients with the higher PAGln levels had higher risks of obstructive IHD and higher coronary lesion complexity [110]. Mechanically, genetic engineering studies followed by microbial transplantation showed that PAGln contribute to the thrombosis potential by accelerating platelet clot formation, calcium release, and responsiveness to multiple agonists. By using multiple genetic and pharmacological screening, PAGln was found to interact with G‐protein‐coupled receptors, in particular adrenergic receptors (ADRs), including α2A, α2B, and β2‐ADRs, which highly present on human platelets. ADRs are crucial for cardiovascular functions and closely related to cardiovascular events [111] and platelet activity [112]. Selective ADR inhibitors can reduce the platelet hyperreactivity induced by PAGln and the acceleration rate of thrombosis in vivo [43]. Similar to TMAO, PAGln and PAGln‐releasing gut microbes appear to be the other potential targets for treating IHD in future efforts.

### Other microbiota‐related metabolites

Among the well‐known microbiota‐related metabolites, two protein‐bound uremic toxins, p‐cresol sulfate (PCS) and indoxyl sulfate (IS), are associated with cardiovascular events [113, 114, 115] and cardiovascular stiffening [116] in patients with chronic kidney disease (CKD). Indeed, CKD patients are often found to display a substantial increase in cardiovascular disease [117]. In the rat CKD model, etiological evidence has been described that PCS and IS may, via activating the coagulation and pro‐inflammatory pathways, contribute to the onset and development of calcification in the vessel wall [118] (Table 1). However, it is important to note that conflicting results exist for the absence in associations between PCS, IS, and cardiovascular outcomes in patients undergoing hemodialysis [119]. Caution is required in the causal interpretation of PCS and IS in IHD development.

## MICROBIOTA‐BASED THERAPY OF IHD

### Therapy targeting gut microbiota

Considering the high mortality and morality of IHD in modern society, clinical translation of the identified IHD‐specific microbiota‐dependent targets is urgently needed. In response to the arising evidence indicating gut microbiota plays a crucial role in IHD, more and more attention has been paid to the therapeutic strategies targeting gut microbial modulation. In the following section, we will highlight several tools and strategies to modulate gut microbial community and their potential in IHD intervention (Figure 3).

### Fecal microbiota transplantation (FMT)

FMT allows for the nutritional enrichment or depletion to the host microbiota, inhibits the growth of pathogenic bacteria, and regulates the host's immune system by transplanting and recolonizing live functional bacterial community from healthy donors into the patient's gastrointestinal tract [120]. FMT is the most fundamental intervention for intestinal microbiota and it is also an established and widely accepted method for the treatment of recurrent *Clostridium difficile* infection [121]. Although it has been shown that obese individuals who receive FMT from lean donors gained enhanced insulin sensitivity and improved phenotypic parameters related to metabolic syndromes [122], the outcomes are highly varied among studies. For instance, the TMAO levels in individuals with metabolic syndrome are unexpectedly not associated with FMT from a single vegan donor, whereas the gut microbial composition of recipients changes towards that of vegan's, pointing to the importance of big sample size and prolonged follow‐up periods in FMT to get desired effects. In addition to the unremarkable changes in TMAO production upon FMT, recent study also reported that transplanting drug‐resistant *E. coli* led to the death of one patient [123], raising the safety concerns of FMT for clinical use. It must be mentioned that challenges in FMT still exists, including the knowledge about optimal conditions for anaerobic handling of donor stools, the incompatibility between recipients and donors, as well as the instability in the survival and recolonization of donor bacteria in recipients’ intestinal tract.

### Antibiotics

Broad‐spectrum antibiotics are commonly used in early experiments targeting the intestinal microbiota for IHD. In 2004, the study by Conraads et al. [124] evidenced that broad‐spectrum antibiotics reduces the biomarker of systematic inflammation in patients with heart failure, but not specifically focused on clinical symptoms. Galla et al. [125] found that minocycline and vancomycin intervention remarkably increased systolic blood pressure in salt‐sensitive rats and decreased systolic blood pressure in spontaneously hypertensive rats. Rune et al. [126] showed in ApoE‐deficient mice, ampicillin intervention could reduce blood low‐density lipoprotein and very low‐density lipoprotein levels. It is interesting to see in the recent study, the oral administration of broad‐spectrum antibiotics increased the mortality of myocardial infarction murine model [127]. This is contrary to the previously reported results that oral vancomycin or a mixture of streptomycin, neomycin, polymyxin B, and bacitracin can reduce myocardial infarction size and improve cardiac function [128, 129]. In trials on patients, outcomes also vary, where some studies showed beneficial effects of antibiotics on IHD [130], whereas others did not. The 10‐year follow‐up data from the Claricor trial showed an increase in cardiovascular death in patients with stable CHD treated with clarithromycin [131]. Therefore, treating IHD with antibiotics remains controversial. Considering these safety problems and the lack of reliable clinical consequences in many trials, antibiotics should be used with caution in future studies aiming at re‐structuring intestinal microbiome of IHD.

### Probiotics and prebiotics

Probiotics can functionally and compositionally interfere with or modulate intestinal microbiota, subsequently activating the immune system and conferring a health benefit [132]. Common probiotics, which have been widely used in clinical practice include *Lactobacillus* and *Bifidobacterium* [133, 134]. Prebiotics, which can stimulate the activity of probiotics, are substrates selectively utilized by the host microorganisms. Most prebiotics are carbohydrates, which can induce the increase in SCFAs and improve metabolic health [134, 135]. Animal models have already suggested that some probiotics and prebiotics such as *Lactobacilli* and inulin can slow down atherosclerosis. Rats treated with *Lactobacillus plantarum* 299v before coronary artery ligation reduced myocardial infarction area and improved heart function [129]. Mohania et al. [136] found that deposition of cholesterol and TAGs in liver and aorta were significantly reduced in rats fed with probiotic dahi. Another study found that obese volunteers who received 20 g/day of inulin‐propionate ester have reduced pro‐inflammatory interleukin‐8 levels compared with those who received cellulose, whereas inulin had no impact on the systemic inflammatory markers [137]. These observations suggested that probiotics and prebiotics may have therapeutic capacity of reducing hyperlipidemia and diet‐induced hypercholesterolemia. In patients with chronic systolic heart failure that was submitted to a 3‐month daily oral supplementation of *Saccharomyces boulardii* (1 g per day) present an improvement in left ventricular ejection fraction and a reduction on left atrial diameter [138]. Except for the relatively small sample size, the beneficial enlightenment of probiotics on IHD represents a promising therapeutic measure for preventing IHD and its related complications.

### Controlling for diet and medication

Genetic factors and environment are both play a great role in the composition of gut microbiota. Dietary details in published studies are commonly lacking or ignored. In the human cohort, dietary records are difficult to access and highly individualized. One recent study found associations between the composition of gut microbiota and red wine, salt intake. Alcohol consumption frequency itself was robustly associated with differences in the distribution of several microbiota such as *Firmicutes* and *Actinobacteria*. In addition, the consumption of wine and beer or cider was strongly associated with differences in gut microbial composition [139]. Several studies have examined the impacts of diet on intestinal flora and disease by giving mice a high‐salt diet (HSD). The composition of gut microbiota was changed in the HSD mice. Erwinia, *Christensenellaceae* and *Corynebacteriaceae*, increased in HSD mice. In contrast, the quantity of *Anaerostipes* reduced in the HSD mice [140]. In subsequent studies, Bier et al. found seven unique taxa that were significantly associated with blood pressure [141]. There was a significant difference in fecal acetic acid, as well as propionic and isobutyric acids, but not in the butyric acid composition between HSD mice and normal‐diet ones [142]. Dietary control is a very practical way to adjust intestinal microbiota. For example, reducing red meat intake is a feasible and effective method to control TMAO level [143], high‐fiber diet for a short term significantly alters the gut microbiome and reduces constipation.

Medication is another crucial but unignorable confounder of gut microbiota. A recent study has shown that 19 drug groups and their metabolism were associated to the composition of intestinal microbiome [144]. Some antihyperglycemic drugs such as metformin, likely through restructuring the gut microbiome at multi‐taxonomic levels,  as well as regulating various microbial functions, particularly microbial genes encoding metalloproteins or metal transporters, to impact human glucose homeostasis [145]. Statins are widely prescribed in clinic combined with aspirin for synergistically lowering blood atherosclerotic lipoproteins [145], it has been found that, although without accounting for other potential confounders, nor inferring the causality behind the observational outcomes, statin therapy is associated with lower prevalence of Bact2 enterotype [146], which was considered as a pro‐inflammatory gut microbial community type. Despite the findings indicating statins are possible targets for designing drug‐based gut microbial modulations, longitudinal, double‐blinded, randomized, placebo‐control study design adjusting for any potential confounders is required to further elucidate the causal relationship between statin therapy and lower Bact2 prevalence. This strategy is also recommended to be acknowledged for observational microbiome studies in cross‐sectional cohorts for a better clarification on the nature of microbiome‐disease links.

## GUT VIROME: AN “EMERGING STAR” FOR UNDERSTANDING AND TREATING IHD

In the past few years, IHD has been proven to be associated with a variety of viruses such as hepatitis virus [147, 148, 149], human immunodeficiency virus [150], and periodontal viruses such as cytomegalovirus and Epstein‐Barr virus [151]. It remains to be explored whether gut virome alterations contribute to the development of IHD. As an important part of the gut microbiome, the function of the virome has gained more and more attention in recent years. The human gut virome is also known as the phageome, as phages make up the vast majority of it [152]. Roughly, there are 10^9^ virus‐like particles (VLPs) per gram of feces, which is an order of magnitude higher than the total number of bacterial cells [153]. Although the advance of next‐generation sequencing technology enables us to further obtain the relevant sequencing reads of enterovirus [154], many challenges still exist. First, it must be mentioned that the gut virome is highly specific and dynamic among individuals, with scant overlap between healthy subjects [154]. Second, as phages rely on bacteria as their host to grow and function, so most enterovirus groups cannot be evaluated by conventional laboratory methodologies except for high‐throughput isolation of single phage species/strains, which is extremely time‐consuming and labor‐intensive. Currently, the mainstream strategy for profiling gut virome are VLPs DNA sequencing or whole‐community metagenome sequencing that require complex data processing and computational resources, while present viral genome database only covers a small proportion of existing viruses in human intestine [152, 155]. These challenges need to be addressed before a high‐resolution snapshot of gut virome is defined.

In recent years, research examining compositions of intestinal phages in specific type of IHD is gradually emerging despite the technical challenges in profiling the gut virome. A compositional analysis of gut virome where viral sequences were profiled in metegenomes of patients with CHD has resulted in *Virgaviridae* and *Microviridae* as the two dominant types of viruses in the enteric virome of CHD subjects [156]. Compared with the gut virome of healthy individuals, CHD gut virome is characterized by enriched *Virgaviridae* but reduced *Microviridae*; however, the underlying mechnisms remained to be explored. Additionally, Jie et al. [9] have identified a panel of differential bacteriophages that are specifically altered in ACVD, but displaying non‐significance in patients with rheumatoid arthritis, T2D, and obesity. Of interest, the known hosts for ACVD‐specific bacteriophages are dominant by *Enterobacteriaceae* at family level or *Streptococcus* at genus level. In another study of individuals with hypertension, a risk factor for IHD [157], it was found a panel consisting of 32 viruses displayed high discriminative power than that of gut bacteriome for the differentiation between people with hypertension and healthy individuals and prehypertention group.

Compared with the sparse knowledge of gut virome functionality from a genetic point of view, the importance of gut virome from an evolutionary perspective appears to be better clarified. Bacteria–phages coevolution, the reciprocal evolution between bacterial hosts and the phages that live on or infect them, is an important driver of ecological and evolutionary processes in microbial communities. There is growing evidence from both laboratory and natural populations that coevolution can maintain phenotypic and genetic diversity, increase the rate of bacterial and phage evolution and divergence, affect community structure, and shape the evolution of ecologically relevant bacterial traits [158]. In the transkingdom interactions, the specific type of interaction between bacteria and phage can be quickly reflected in host immunity and infectious phenotype [159]. This directly or indirectly promotes genetic and phenotypic divergence, competitions, and cooperations [160, 161]. Indeed, in the transkingdom interaction analysis between gut virome and bacteriome in participants with hypertension, it was shown that hypertension group has higher number of linkages between viruses and bacteria in comparison with the healthy controls and prehypertension subjects. Gut phage is also considered a potential therapeutic target due to its lytic interaction with bacteria. In a recent study, Yi et al. [162] investigated the therapeutic effects of bacteriophages that target cytolytic *E. faecalis* by using humanized mice that were colonized with bacteria from the faeces of patients with alcoholic hepatitis. They showed that these phages decrease cytolysin in the liver and abolish ethanol‐induced liver disease in humanized mice [162]. Torben et al. [163] found that faecal virome transplantation cases showed significant weight loss in murine models. They supposed that faecal virome transplantation can ameliorate obesity and diabetes by changing the gut microbiota [163]. Considering IHD is closely related to infection and immunity, it is reasonable to hypothesize gut virome may play an important inducing role in the occurrence of IHD, and whether bacteriophages are potent in treating IHD by infecting IHD‐enriched specific pathogens remains to be further explored.

## FUTURE PERSPECTIVES

Despite the immense alterations in taxonomic and functional potentials of gut microbiome in IHD, major reports on IHD microbiome studies appear to be observational, association‐based, and lacking specificity. With the focus on the influence of the gut microbiome on the overall functional readouts of IHD, much still needs to be learned. It is of great importance to decipher and annotate hundreds of yet non‐annotated chemicals in the metabolome of various biological fluids as well as their origins (solely host, microbial, and dietary origin, or combined origin). As most studies are based on cross‐sectional cohorts, sparse information of the microbial dynamics in IHD is given, either relevant bioinformatic algorithms predicting the short‐ or long‐term dynamics of gut microbiome, or longitudinal data is required to fill this gap. Last but not least, little of the novel knowledge is validated or has maturated to gain potential in being translated to guide clinical practice of IHD intervention. Future, with the progress in sequencing‐based and culture‐based gut microorganism surveys combined with mechanistic exploitations of the gut bacteriome, phageome, and virome, our knowledge towards the interactions within the global gut microbial system will be exponentially expanded.

## CONCLUSIONS

As discussed in various literatures linking gut microbial features and IHD, human gut microbiota‐related metabolites appear as major mediators. In this review, we have not only summarized the various gut microbial taxa that are linked to multiple IHD stages, but also highlighted the up‐to‐date knowledges of microbial metabolites and their potential roles in mediating the impact of gut microbial alterations on progression of IHDs. Last but not least, we highlighted the gut virome as an additional dimension for mechanistic dissections and understandings of IHD.

## AUTHOR CONTRIBUTIONS

Yong Fan and Hanbin Cui contributed to the overall conceptualization. Yong Fan and Jiajun Ying contributed equally to the writing and discussion of the main content of this manuscript. All authors have read and approved the final manuscript.

## CONFLICT OF INTEREST STATEMENT

The authors declare no conflict of interest.

## Supporting information

Supporting information.

## Data Availability

This manuscript does not generate any code or data.
